# Gamma-Aminobutyric Acid (GABA) as a Defense Booster for Wheat against Leaf Rust Pathogen (*Puccinia triticina*)

**DOI:** 10.3390/plants13192792

**Published:** 2024-10-05

**Authors:** Hala Badr Khalil, Abdullah Mohsen Lutfi, Ahmed Reyad Sayed, Mohamed Tharwat Mahmoud, Salah Abdelfatah Mostafa, Zeyad Ahmed Ibrahim, Asmaa A. Sharf-Eldin, Mohamed A. Abou-Zeid, Mohamed F. M. Ibrahim, Marian Thabet

**Affiliations:** 1 Department of Biological Sciences, College of Science, King Faisal University, P.O. Box 380, Al-Ahsa 31982, Saudi Arabia; 2 Department of Genetics, Faculty of Agriculture, Ain Shams University, Cairo 11241, Egypt; 3 Biotechnology Program, Faculty of Agriculture, Ain Shams University, Cairo 11241, Egypt; abood.lutfy2000@gmail.com (A.M.L.); riadahmed553@gmail.com (A.R.S.); mohamedtharwat9th@gmail.com (M.T.M.); salah.mostafa883@gmail.com (S.A.M.); zeyadsokkar100@gmail.com (Z.A.I.); 4 Department of Agricultural Botany, Faculty of Agriculture, Ain Shams University, Cairo 11566, Egypt; asmaa.ahmed@agr.asu.edu.eg (A.A.S.-E.); ibrahim_mfm@agr.asu.edu.eg (M.F.M.I.); 5 Wheat Diseases Research Department, Plant Pathology Research Institute, Agricultural Research Center, Giza 12619, Egypt; m.abouzeid@arc.sci.eg; 6 Department of Plant Pathology, Faculty of Agriculture, Ain Shams University, Cairo 11241, Egypt; marianshokry@agr.asu.edu.eg

**Keywords:** gamma-aminobutyric acid, *Puccinia triticina*, *Triticum aestivum*, leaf rust, wheat, reactive oxygen species, catalase, peroxidase

## Abstract

Wheat leaf rust, caused by *Puccinia triticina*, poses a growing threat to global wheat production, necessitating alternative strategies for effective disease management. This study investigated the potential of gamma-aminobutyric acid (GABA) to enhance resistance to leaf rust in two wheat cultivars: the susceptible Morocco and moderately resistant Sakha 94 cultivar. Our findings revealed that GABA significantly improved resistance in both cultivars to *P. triticina*, particularly in Morocco, by mitigating disease severity and reducing pustule density and size while extending both incubation and latent periods. This study assessed the effectiveness of two GABA application methods: plants received 1 mM GABA treatment, as a foliar spray, twenty-four hours prior to infection (pre-GABA), and plants received 1 mM GABA treatment both 24 h before and after infection (pre-/post-GABA), with the latter yielding significantly better results in reducing infection severity and improving plant resilience. Additionally, GABA application influenced stomatal behavior, promoting closure that may enhance resilience against leaf rust. GABA application on plants also modulated the production of reactive oxygen species (ROS). This led to a stronger oxidative burst in both susceptible and moderately resistant cultivars. GABA increased O_2_^●−^ levels in guard cells and surrounding stomata, enhancing stomatal closure and the hypersensitive response. GABA enhanced the accumulation of soluble phenols and increased the activity of key antioxidant enzymes, catalase (CAT) and peroxidase (POX), which are vital for managing oxidative stress. To the best of our knowledge, this investigation represents the first report into the impact of GABA on wheat leaf rust disease.

## 1. Introduction

Wheat, *Triticum aestivum*, occupies a pivotal position in the modern diet, supplying a significant portion of the world’s caloric intake. As one of the “big three” cereal crops alongside rice and maize, wheat serves as a staple food for billions of people, providing essential carbohydrates, proteins, and micronutrients necessary for human health and well-being [1]. It plays a vital role in livestock feed, further amplifying its impact on food systems. Understanding the dynamics of wheat production is paramount for ensuring food security and sustainable agricultural development globally in an era characterized by population growth, shifting dietary preferences, and economic globalization [2].

Rust diseases pose a significant threat to global wheat production, comprising three pathogens: leaf, stripe, and stem rusts, collectively known as “rusts.” These fungal diseases, caused by various species of the *Puccinia* genus, have plagued wheat crops for centuries, wreaking havoc on yields and challenging agricultural sustainability [3]. Rust epidemics result in substantial yearly yield losses, with affected crops exhibiting diminished productivity due to premature leaf senescence, reduced photosynthetic capacity, and impaired nutrient uptake [4]. Moreover, the quality of harvested grain suffers, as rust-infected wheat often exhibits lower protein content, compromised gluten strength, and increased susceptibility to pre-harvest sprouting and mycotoxin contamination. The economic toll on farmers is profound, with losses stemming from decreased marketable yield, increased input costs for fungicides and disease management, and reduced market value due to inferior grain quality. As such, the menace of rust diseases looms large over wheat-producing regions, underscoring the urgent need for comprehensive strategies to mitigate their impact and safeguard global food security [5]. The adaptability of rust pathogens, coupled with the emergence of new virulent strains and changing climatic patterns, exacerbates the challenge of rust management for farmers and researchers alike [6].

Leaf rust disease, caused by the fungal pathogen *Puccinia triticina*, unfolds with a distinct set of symptoms and insidious modes of spread within fields. Initially, the fungus forms orange-colored pustules erupting on the upper surfaces of wheat leaves [7]. These pustules gradually enlarge and merge, forming extensive lesions that compromise the structural integrity of the leaves and hinder their ability to photosynthesize effectively. As temperatures moderate and humidity levels rise, conditions become increasingly favorable for the proliferation of leaf rust, enabling the pathogen to spread rapidly throughout wheat fields [8,9]. Susceptible wheat cultivars, particularly those lacking robust resistance genes, are at heightened risk of succumbing to leaf rust under these conducive environmental conditions [10,11]. Ultimately, the cumulative effects of leaf rust infection culminate in diminished yield potential and compromised grain quality, posing significant challenges to farmers striving to maintain agricultural productivity and meet the demands of a growing population [7,8].

Sakha 94, a wheat cultivar renowned for its durable resistance against several races of leaf rust fungus, exemplifies a significant advancement in breeding for disease resistance. Studies have identified specific genetic loci in Sakha 94 associated with resistance genes that trigger defense responses upon pathogen recognition [12]. Sakha 94, developed in Egypt, emerged from a breeding program focused on enhancing wheat varieties’ resilience to biotic stresses, particularly fungal diseases like leaf rust. It bred for adaptability to local environmental conditions and resistance traits [12]. Recent studies have evaluated various wheat cultivars, including Sakha 94, Misr 3, and Gemmeiza 12, for their response to leaf rust at different growth stages. Biochemical, histological, and genetic analyses have demonstrated that Sakha 94, along with other cultivars, like Misr 3, has moderate resistance at the different growth stages, including seedling and mature plants [13]. Wheat leaf rust was responsible for the extinction of numerous cultivars in Egypt, including Giza-139, Mexipak 69, Super X, Chenab 70, Giza 158, and Giza 160, due to their susceptibility to leaf rust under field conditions. Moreover, some wheat genotypes were discarded very shortly after their release, such as Giza 139 [14,15]. However, susceptibility to leaf rust remains a significant concern, prompting continued research into genetic markers and agronomic practices aimed at bolstering resistance while maintaining yield stability in diverse agroecological zones.

Managing leaf rust disease in wheat presents a multifaceted challenge that requires a comprehensive approach that integrates various strategies. Current management tactics include genetic resistance breeding, where wheat cultivars with inherent resistance to leaf rust are developed through selective breeding programs [16]. Additionally, fungicide applications serve as a crucial tool for controlling leaf rust outbreaks, offering immediate protection to susceptible crops [8]. Cultural practices, such as crop rotation and tillage management, also play a role in reducing the spread of leaf rust by disrupting the pathogen’s life cycle. However, despite these efforts, managing leaf rust remains fraught with challenges. One significant obstacle is the continual evolution of new rust strains with heightened virulence, rendering previously resistant cultivars vulnerable to infection [17,18]. However, wheat cultivars that depend on race-specific resistance often lose effectiveness within a few years due to the strong selection pressure for virulent leaf rust races. Moreover, the widespread cultivation of susceptible cultivars allows a large rust population to thrive, creating a reservoir for mutation and selection [19]. Furthermore, the indiscriminate use of fungicides has led to the development of resistance in rust populations, diminishing the effectiveness of chemical control measures. Addressing these challenges necessitates ongoing research and innovation to develop resilient wheat varieties, sustainable fungicide management strategies, and integrated pest management practices tailored to the dynamic nature of leaf rust epidemiology.

Finding sustainable solutions for leaf rust disease resistance in wheat requires a holistic approach that addresses the complex interplay of genetic, agronomic, and ecological factors. One promising avenue is the development of wheat cultivars with durable resistance traits through advanced breeding techniques, incorporating multiple resistance genes to enhance effectiveness and longevity [16]. Moreover, fostering genetic diversity within cultivated wheat populations can bolster resilience to evolving rust pathogens. Integrated pest management strategies, integrating resistant cultivars with cultural practices such as crop rotation and residue management, can help suppress rust populations and mitigate disease pressure [20,21].

Gamma-aminobutyric acid (GABA) emerges as a multifaceted non-protein amino acid with pivotal physiological roles in plants, encompassing stress response and defense mechanisms against pathogens [22]. Previous research elucidates GABA’s involvement in modulating plant immunity and enhancing resistance to a spectrum of biotic stresses [23]. Through its regulatory functions, GABA orchestrates intricate signaling pathways that bolster plant defenses, activate stress-responsive genes, and fine-tune metabolic processes to mitigate the impact of pathogen attacks [24]. In plants, the GABA transporters aluminum-activated malate transporter (ALMT) and GABA transporter (GAT) are key players in the regulation of GABA transport across cellular membranes [25]. ALMT, specifically ALMT1, originally identified for its role in aluminum tolerance by mediating malate efflux, has also been found to transport GABA [26]. This transporter is crucial for maintaining cellular GABA levels and responding to environmental stresses such as aluminum toxicity. GAT, on the other hand, is a dedicated GABA transporter responsible for the uptake of GABA from the extracellular environment into the cytoplasm of plant cells [27]. Both ALMT and GAT play essential roles in regulating GABA homeostasis, thereby influencing various physiological processes, including stress responses and metabolic regulation [28]. Understanding the functions and regulatory mechanisms of these transporters provides valuable insights into the broader roles of GABA in plant biology and offers potential avenues for improving stress tolerance and crop productivity through targeted manipulation of GABA transport pathways.

Building upon this foundation, a hypothesis emerges suggesting that exogenous application of GABA could serve as a potent strategy to stimulate wheat-plant defense mechanisms and fortify resistance against the insidious leaf rust pathogen. With its established role in modulating plant immunity and stress responses, GABA holds promise as a bioactive compound capable of priming the plant’s innate defense mechanisms [22]. By exogenously applying GABA to wheat plants, researchers aim to activate defense-related signaling pathways, induce the expression of pathogenesis-related genes, and enhance the synthesis of defense compounds such as antioxidants [29]. This priming effect is anticipated to confer a heightened state of readiness in wheat plants, enabling them to mount a more robust and effective defense response upon encountering the leaf rust pathogen.

Given the escalating threat posed by leaf rust disease to wheat production and the inherent limitations of conventional management approaches, there is an urgent need to explore alternative strategies for sustainable disease control. For this, the experiment aimed to assess the effectiveness of GABA in bolstering resistance to leaf rust in Sakha 94 and Morocco, the moderately resistant and susceptible wheat cultivars to leaf rust. Symptom records, collected based on infection types (ITs) observed in wheat plants, provided valuable data on disease progression over time. The first leaves of wheat seedlings were visually inspected at regular intervals post-inoculation, revealing distinct patterns of rust symptom development. Samples were collected for histological examination, revealing the presence of reactive oxygen species (ROS), including superoxide anion (O_2_^●−^) and hydrogen peroxide (H_2_O_2_), within the wheat leaf tissue due to oxidative stress induced by the fungal pathogen infection. A physiological analysis involving phenol content, as well as catalase (CAT) and peroxidase (POX), enzymes elucidated the plant’s antioxidant defense mechanisms in response to GABA treatment.

## 2. Results

### 2.1. GABA Enhanced Wheat Resistance to Leaf Rust Fungal Infection

**Figure 1:**
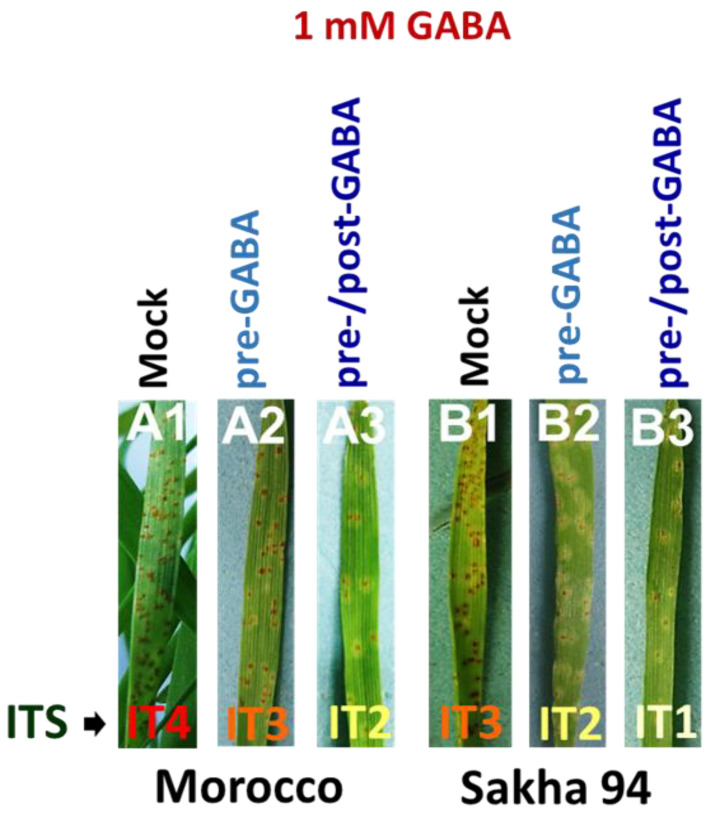
Impact of exogenous GABA applied to wheat-seedling leaves on the symptoms of leaf rust disease. The infection types induced by *Pt* race PTKTT on both the susceptible, Morocco (A1–A3), and moderately resistant, Sakha 94 (B1–B3), wheat cultivars were recorded and photographed. The treatments encompass mock-treated plants (A1 and B1), pre-GABA-treated plants (A2 and B2), and pre-/post-GABA-treated plants (A3 and B3). GABA treatment altered the infection types (ITs) of Morocco cultivar as follows: IT4 for mock (A1), IT3 for pre-GABA (A2), and IT2 for pre-/post-GABA (A3). On Sakha 94, GABA treatment shifted the ITs: IT3 for mock (B1), IT2 for pre-GABA (B2), and IT1 for pre-/post-GABA (B3). The mock-treated plants, H_2_O foliar-sprayed plants; pre-GABA, plants received 1 mM GABA treatment, as a foliar spray, twenty-four hours prior to infection; pre-/post-GABA, plants received 1 mM GABA treatment both 24 h before and after infection.

The foliar spraying of GABA on wheat seedlings significantly altered the infection type scores observed in both susceptible and moderately resistant cultivars. For the susceptible cultivar (Morocco), the mock-treated plants (H_2_O foliar-sprayed plants either infected or uninfected by leaf rust) exhibited an infection type 4 (IT4) (Figure 1), indicating high susceptibility to *P. triticina* (*Pt*). In contrast, Morocco cultivar plants that received 1 mM GABA treatment, in the form of a foliar spray, twenty-four hours prior to infection (pre-GABA-treated plants) showed reduced symptom records of IT3, demonstrating an intermediate level of resistance. Furthermore, the plants that received 1 mM GABA treatment both 24 h before and after infection (pre-/post-GABA-treated plants) displayed an even lower IT2, indicating enhanced resistance compared to the mock treatment. Similarly, for the moderately resistant cultivar (Sakha 94), the mock-treated plants showed an IT3 (Figure 1), reflecting a moderate level of resistance. The resistance of pre-GABA-treated plants was further improved, resulting in an IT2. The most significant improvement was for the Sakha 94 cultivar observed in the plants treated with GABA both before and after infection, which exhibited an IT1, signifying a high level of resistance to *Pt*.

The scoring and infection analyses of the *Pt* on the Morocco and Sakha 94 wheat-seedling first leaves unveiled a significant reduction in the severity of leaf rust disease symptoms following the application of GABA. In the Morocco cultivar, GABA treatment led to a decrease in pustule density from 32.88 pustules/cm^2^ in the mock-treated plants to 24 pustules/ cm^2^ in the pre-GABA-treated plants and 12.06 pustules/cm^2^ in the pre-/post-GABA-treated plants, accompanied by a reduction in pustule size from 0.947 mm^2^ in the mock-treated plants to 0.586 mm^2^ in the pre-GABA-treated plants and 0.356 mm^2^ in the pre-/post-GABA-treated plants (Table 1). Additionally, changes in disease incubation and latent periods were observed in favor of the pre-/post-GABA-treated plants in 11.18 dai and 15.95 dai compared to the pre-GABA-treated plants in 7.78 dai and 13.15 days after inoculation (dai), as well as mock-treated plants in 6.25 dai and 11.27 dai, respectively.

On the other hand, in the Sakha 94 cultivar, the impact of GABA treatment was milder compared to the susceptible cultivar. While reductions in leaf rust disease symptoms were observed, they were less pronounced. Specifically, GABA treatment led to a modest decrease in pustule density from 23.42 pustules/cm^2^ in the mock-treated plants to 17.83 pustules/cm^2^ in the pre-GABA-treated plants and 9.16 pustules/cm^2^ in the pre-/post-GABA-treated plants. Similarly, there was a slight reduction in pustule size from 0.743 mm^2^ in the mock-treated plants to 0.393 mm^2^ in the pre-GABA-treated plants and 0.253 mm^2^ in the pre-/post-GABA-treated plants. Changes in disease incubation and latent periods were also observed, albeit to a lesser extent, favoring the pre-/post-GABA-treated plants in 13.2 dai and 20.01 dai compared to the pre-GABA-treated plants in 11.18 and 15.3 dai and mock-treated plants in 8.58 and 14.22 dai, respectively.

### 2.2. GABA-Mediated Stomatal Closure in Wheat Leaves under Dark Conditions

**Figure 2:**
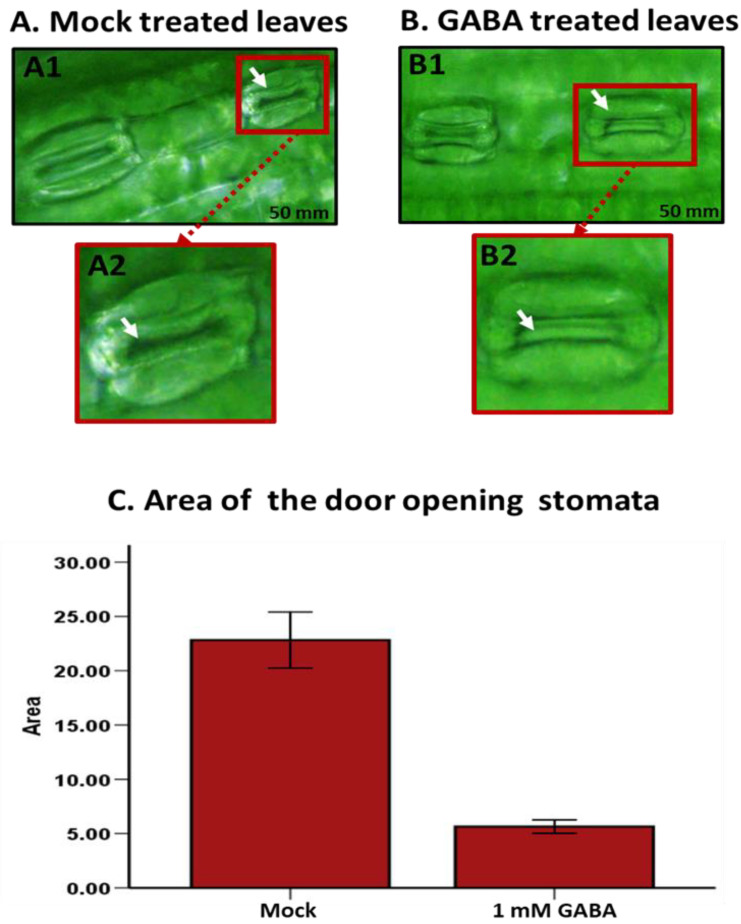
GABA application induced stomatal closure in uninfected wheat-plant leaves. After a 24-h period of darkness to stimulate stomatal opening, GABA-treated leaves exhibited marked closure, while mock-treated leaves remained predominantly open. (**A1**,**B1**) Screening of leaves treated with a 1 mM GABA as a foliar spray, three hours post-treatment, confirmed GABA’s efficacy in inducing stomatal closure under dark conditions. Panels (**A2**,**B2**) provide a zoomed-in magnification. (**C**) The average of stomatal aperture area was 23.28 mm^2^ under mock treatment and 5.07 mm^2^ under GABA treatment, indicating significant stomatal closure with GABA application. The screening and analysis included 20 stomata for each of the mock and GABA treatments. White arrows represent stomata gate. Scale bar = 50 μm.

The application of GABA resulted in a striking response in the wheat-seedling leaves, notably inducing pronounced stomatal closure. In an effort to assess the extent of this effect, a 24-ho period of darkness was employed to stimulate stomatal opening in wheat plants. Following this period of darkness, GABA-treated plant leaves exhibited significant closure of stomata compared to leaves treated with a mock solution, where stomata predominantly remained open (Figure 2A,B). Screening of wheat leaves treated with 1 mM GABA spray and inspected three hours post-treatment demonstrated the efficacy of GABA in inducing stomatal closure under dark conditions. Quantitative analysis of stomatal aperture area measurements, facilitated by ImageJ analysis software (version 1.54), revealed a marked decrease in the stomatal opening pore in the GABA-treated wheat leaves relative to the mock-treated cohort. Statistical scrutiny confirmed a significant disparity in stomatal closure between the two treatments (Figure 2C). By quantifying the relative area of stomatal aperture compared to the total stomatal area, we inferred changes in stomatal opening. The inclusion of 20 stomata per treatment group ensures the robustness of these results. Under mock treatment conditions, the mean area of stomatal apertures was recorded at 23.28 mm^2^. Conversely, GABA treatment resulted in a considerable reduction in aperture size, with an average area of 5.07 mm^2^, indicating substantial closure of most stomata. Our statistical analysis of the t-test revealed significant disparities between the two treatment groups, underscoring the efficacy of GABA in modulating stomatal apertures. These findings not only highlight the potency of GABA as a regulator of stomatal behavior but also shed light on its potential applications in enhancing plant responses to environmental cues.

### 2.3. Accumulation of ROS Enhanced by GABA Treatment

**Figure 3:**
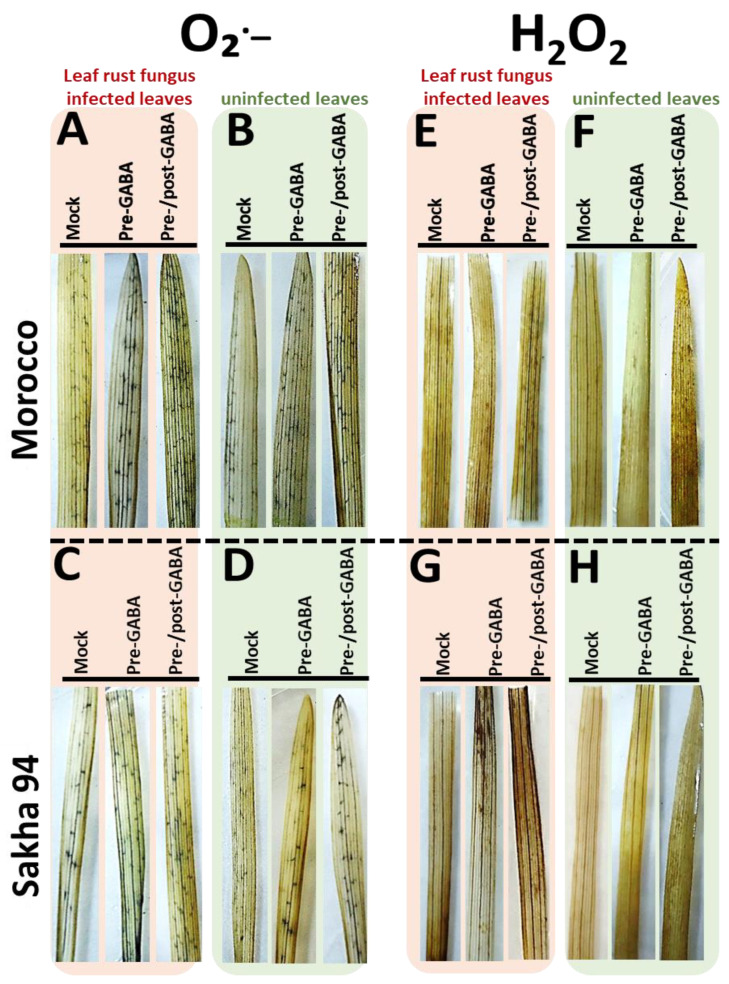
Localization of superoxide anion (O_2_^●−^) and hydrogen peroxide (H_2_O_2_) on the first leaves of Morocco and Sakha 94, the susceptible and moderately resistant wheat cultivars treated with GABA. The experiment involved two panels, O_2_^●−^ (**A**–**D**) and H_2_O_2_ (**E**–**H**), for the two cultivars, Morocco and Sakha 94, and different treatments: (1) mock-treated (water-sprayed leaves); (2) pre-GABA-treated plant (plants received 1 mM GABA treatment, as a foliar spray, twenty-four hours prior to infection); and (3) pre-/post-GABA-treated plants (plants received 1 mM GABA treatment, as a foliar spray, twenty-four hours before and after the infection). Samples were collected 3 dai.

**Figure 4:**
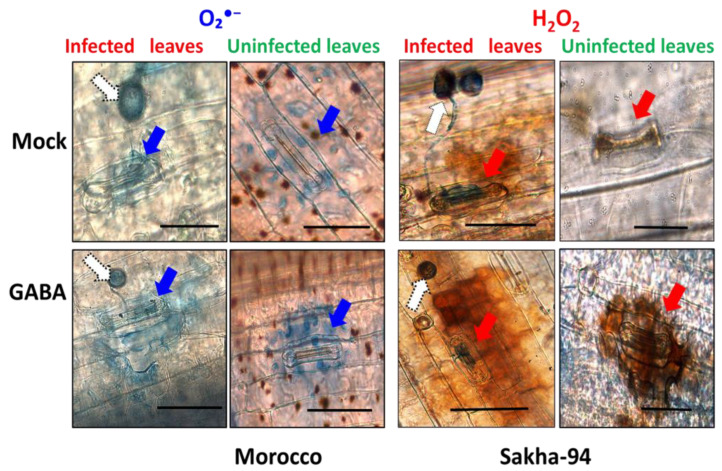
Microscopic analysis of Morocco and Sakha 94 wheat epidermal leaves, illustrating the localization of O_2_^●−^ and H_2_O_2_ surrounding stomatal cells. Microscopic images in two panels. The first panel depicts the localization of O_2_^●−^ in mock and pre-/post-GABA-treated Morocco plants for *Pt*-infected and -uninfected plants, respectively. Blue arrows indicate regions contributing to stomatal closure and the hypersensitive response. The second panel reveals the distribution of H_2_O_2_ in mock and pre-/post-GABA-treated Sakha 94 plants for *Pt*-infected and -uninfected plants, respectively. Red arrows refer to O_2_^●−^ reaction. Red arrows indicate regions contributing to stomatal closure and the H_2_O_2_ hypersensitive response. White arrows show *Pt* germ pod. Scale bar = 20 μm.

GABA treatment increased the levels of reactive oxygen species (ROS), particularly in the pre-/post-GABA application, which contributed to an enhanced oxidative burst in both susceptible and moderately resistant wheat cultivars in response to leaf rust fungal infection. Herein, the accumulation of O_2_^●−^ was detected generally in guard cells, surrounding stomata, and contributing to stomatal closure and reactive response (Figure 3A–D). GABA treatment increased the accumulation of superoxide anion (O_2_^●−^) expression in tested wheat cultivars when compared to mock-treated plants. In addition, upon infection with Pt, the susceptible Morocco cultivar exhibited a higher accumulation of O_2_^●−^ (Figure 3A,B) compared to Sakha 94, the moderately resistant cultivar (Figure 3C,D). Interestingly, the accumulation of O_2_^●−^ was also detected microscopely and found surrounding guard cells. The microscopy analyses of Morocco cultivar epidermal leaves revealed the localization of superoxide (O_2_^●−^) as blue spots nearby stomata (Figure 4). The distributions of O_2_^●−^ in mock- and pre-/post-GABA-treated plants as infected and uninfected indicated the regions contributing to stomatal closure. This observation aligns with findings that exogenous GABA can modulate oxidative stress responses in plants, promoting resistance against various stressors, including pathogen attacks.

Here, we also observed consistent production of hydrogen peroxide (H_2_O_2_) in both tested cultivars, indicating an inherent constitutive defense mechanism. The accumulation of H_2_O_2_ was detected in guard and mesophyll cells, contributing to stomatal closure and the hypersensitive response (Figure 3E–H). Notably, the infected moderately resistant cultivar demonstrated markedly elevated levels of H_2_O_2_ compared to its susceptible counterparts, suggesting a robust and enhanced defense response. Moreover, the study revealed intriguing observations of GABA application in modulating H_2_O_2_ dynamics. Specifically, pre-/post-GABA-treated infected Sakha 94 plants revealed a pronounced increase in H_2_O_2_ (Figure 3E), whereas there was a modest augmentation in H_2_O_2_ levels within the infected Morocco cultivar (Figure 3G). The accumulation of H_2_O_2_ was also microscopically detected in guard cells and mesophyll cells, contributing to Sakha 94 plant defense (Figure 4). Notably, GABA-treated plants exhibited increased production of H_2_O_2_ around the stomata, highlighting the differential responses of Sakha 94 cultivar to exogenous signaling molecules. These findings suggest that O_2_^●−^ and H_2_O_2_ play crucial roles in mediating oxidative stress responses and plant resilience against pathogens, particularly in the context of GABA-induced priming of defense pathways.

### 2.4. GABA Enhancing Phenolic Accumulation in Plants

**Figure 5:**
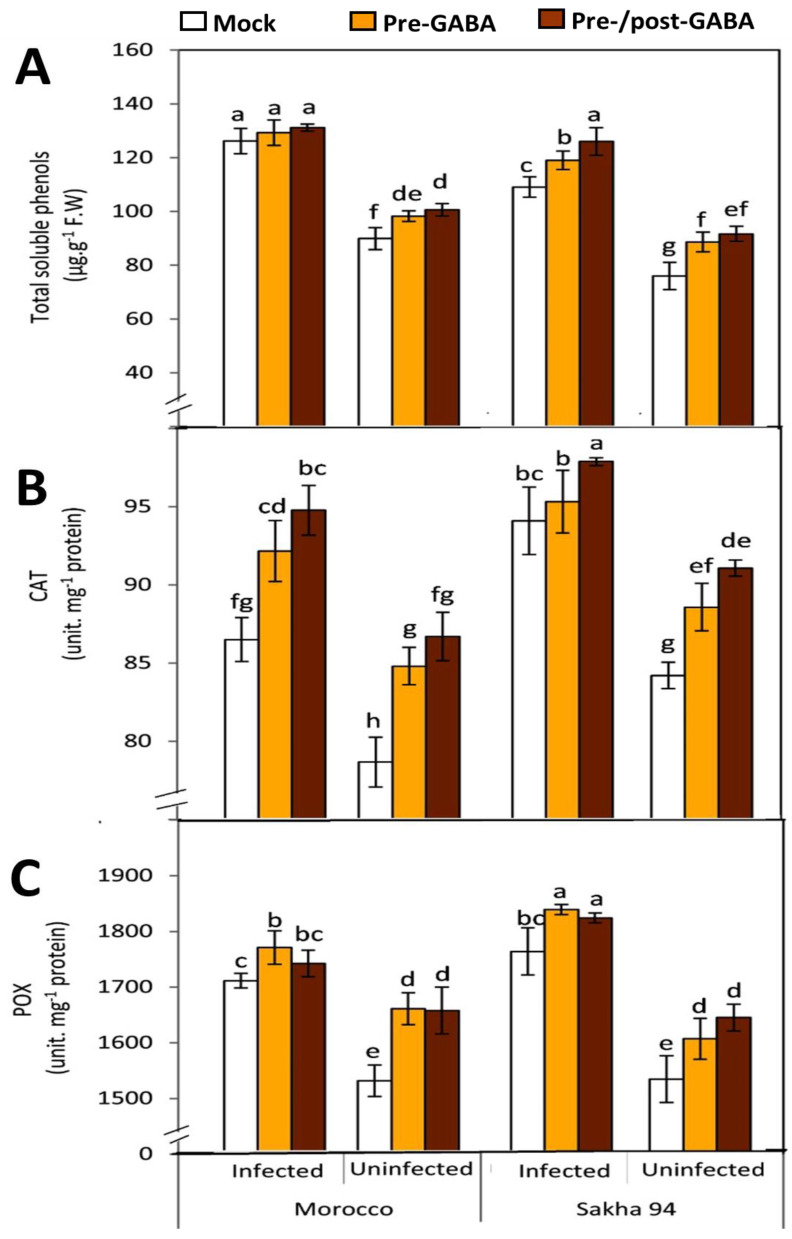
Effects of GABA treatment on the levels of total soluble phenols, catalase activity (CAT), and peroxidase activity (POX) in infected and uninfected Morocco and Sakha 94 wheat cultivars. (**A**) Total soluble phenols were significantly elevated in infected plants compared to uninfected controls in both cultivars. (**B**) CAT activity increased in infected plants, particularly in Sakha 94, where GABA treatment further enhanced CAT activity. (**C**) POX activity was also elevated in infected plants across both cultivars, with GABA treatment leading to increased POX activity in both infected and uninfected plants. White bars represent mock-treated (water-sprayed leaves); yellow bars indicate pre-GABA-treated plant (plants received 1 mM GABA treatment, as a foliar spray, twenty-four hours prior to infection); brown bars present pre-/post-GABA-treated plants (plants received 1 mM GABA treatment, as a foliar spray, twenty-four hours before and after the infection). Different letters above the bars denote statistically significant differences (*p* < 0.05) among treatments within each group, as determined by the Tukey test. Error bars represent the standard error of the mean.

The analysis of total phenols revealed that GABA-treated plants from both the Morocco and Sakha 94 wheat cultivars exhibited elevated levels of soluble phenols. In general, the infection with *Pt* significantly increased phenolic content across both cultivars (Figure 5A). In Morocco cultivars, no statistically significant differences were observed between mock-treated and GABA-treated infected plants, with phenol concentrations ranging from approximately 120 to 140 µg/g fresh weight (FW). Conversely, uninfected plants demonstrated significantly lower soluble phenol levels, ranging from approximately 90 to 110 µg/g FW. Notably, slightly higher phenolic concentrations were observed in pre-GABA- and pre-/post-GABA-treated plants compared to mock-treated plants. In the Sakha 94 cultivar, infected plants also showed increased levels of soluble phenols, around 120–130 µg/g. Significant differences among the treatments were noted. In contrast, uninfected plants had the lowest phenolic content, with mock-treated plants measuring around 80 µg/g “g,” while pre-GABA- and pre-/post-GABA-treated plants exhibited slightly higher levels.

### 2.5. Antioxidant Activation as a Response to GABA Treatment

#### 2.5.1. Catalase (CAT)

Exogenous application of GABA significantly enhanced CAT enzyme activity in both wheat cultivars, with a more pronounced increase observed in infected Sakha 94 plants (Figure 5B). Infection with *Pt* led to elevated CAT activity in both the Morocco and Sakha 94 cultivars. In the infected Morocco cultivar, pre-/post-GABA-treated plants exhibited the highest CAT activity, approximately 95 units/mg protein, in contrast to lower activity levels observed in pre-GABA- and mock-treated plants. Uninfected Morocco plants demonstrated significantly reduced CAT activity, with values ranging from approximately 75 to 88 u/mg. Similarly, infected Sakha 94 plants exhibited high CAT activity levels between 95 and 99 u/mg, with the highest activity recorded in pre-/post-GABA-treated plants. Uninfected Sakha 94 plants also showed lower CAT activity, with values ranging from approximately 85 to 92 u/mg.

#### 2.5.2. Peroxidase (POX)

GABA treatment significantly increased POX activity in both wheat cultivars, paralleling the effects observed on CAT activity. The analysis of POX activity indicated that infected plants from both the Morocco and Sakha 94 cultivars exhibited markedly elevated levels of POX activity (Figure 5C). Specifically, infected Sakha 94 plants demonstrated POX activity values ranging from approximately 1750 to 1850 u/mg, with the highest values recorded in pre-/post-GABA- and pre-GABA-treated plants. In contrast, uninfected Sakha 94 and Morocco plants exhibited significantly lower POX activity, with values between 1550 and 1700 u/mg for GABA-treated plants and mock-treated plants. These results suggest that GABA application effectively enhances the antioxidant enzyme activities in response to pathogen infection, contributing to the overall resilience of the wheat cultivars.

## 3. Discussion

As the prevalence of wheat leaf rust disease, caused by *P. triticina*, continues to increase, the limitations of conventional disease management strategies are becoming more evident. Developing sustainable solutions for leaf rust resistance in wheat requires a comprehensive approach that addresses the complex interplay of genetic, agronomic, and ecological factors. This endeavor faces several challenges, including the rapid evolution of pathogen races, the necessity for effective and adaptable breeding strategies, and the implementation of widely adoptable agronomic practices [30]. Given these escalating challenges, it is crucial to explore alternative approaches for sustainable control. In this investigation, we evaluated the efficacy of GABA in enhancing resistance to leaf rust in wheat cultivars with varying levels of inherent resistance. Specifically, we focused on the susceptible cultivar Morocco and the moderately resistant cultivar Sakha 94. Our findings contribute to a growing body of evidence supporting the potential of GABA as a biostimulant for improving plant defense. A completely randomized design with three replicates was employed to assess the impact of exogenous GABA treatment on both infected and uninfected plants with different genetic backgrounds challenged with *Pt*. The results highlight the significant impact of exogenous GABA on wheat resistance to leaf rust infection, demonstrating a clear enhancement in both susceptible and moderately resistant cultivars. In the susceptible wheat cultivar, GABA treatment significantly improved resistance to leaf rust. While mock-treated plants exhibited high susceptibility (IT4), pre-GABA-treated plants reduced the infection type to IT3, indicating intermediate resistance (Figure 1). The most pronounced resistance was observed in pre-/post-GABA-treated plants, showing an IT2. Moreover, exogenous GABA also led to a reduction in the pustule densities and sizes (Table 1). Additionally, it extended the incubation and latent periods of the disease, suggesting a delay in disease progression. These results underscore GABA’s potential in enhancing resistance and controlling leaf rust in susceptible wheat cultivars. In Sakha 94 cultivar, GABA treatment also improved disease resistance, though less dramatically than in the susceptible cultivar. Mock-treated plants had an infection type of IT3, while the infection type of pre-GABA-treated plants was reduced to IT2. The most significant resistance was achieved with pre-/post-GABA-treated plants, resulting in an IT1. Although reductions in pustule densities and sizes were more modest compared to the Morocco cultivar, exogenous GABA still extended the incubation and latent periods of the disease, indicating enhanced resistance. Recent advances in understanding plant interactions with biotic stressors, highlighting GABA’s involvement in modulating plant immunity and bolstering resistance against, have been thoroughly reported [23,24,31,32,33]. For wheat, GABA has shown significant potential in enhancing resistance to fungal diseases. For instance, GABA treatment can effectively bolster the plant’s defense mechanisms against pathogens such as *Stagonospora nodorum*, the causal agent of stagonospora blotch [34]. In addition, GABA application has been demonstrated to reduce the disease severity of strip rust [35]. To the best of our knowledge, our findings represent the first report on the impact of GABA on leaf rust disease in wheat. This suggests that GABA can be a valuable tool for managing leaf rust in wheat, with potential benefits across different cultivars.

The application of GABA in wheat plants significantly influences stomatal behavior, particularly inducing pronounced stomatal closure. Here, wheat leaves sprayed with GABA and assessed three hours post-treatment showed marked stomatal closure under dark conditions (Figure 2). In addition, our quantitative analysis revealed a significant decrease in stomatal aperture in GABA-treated leaves compared to controls. This finding suggests that GABA has potential as a biostimulant to enhance plant responses to environmental cues by regulating stomatal closure, which may improve resilience to biotic and abiotic stresses. Recent studies have highlighted the significant impact of GABA on stomatal regulation in various plant species, including Arabidopsis [36,37,38], maize [39], wheat [40], hordeum [41], bean [42], and rice [43]. In our investigation, GABA treatment was observed to induce significant stomatal closure, thereby reducing the risk of fungal infections, such as leaf rust. GABA plays a crucial role in stomatal regulation through its metabolism and signaling pathways. Synthesized from glutamate by glutamate decarboxylase (GAD), GABA accumulation is often triggered as a stress response [38,40,43]. This accumulation enhances the production of ROS in guard cells, leading to stomatal closure, which is essential for minimizing water loss during stress conditions [25,26].

Pathogens often trigger the accumulation of ROS in plants as part of the plant’s defense response. ROS include O_2_^●−^ and H_2_O_2_ and play a crucial role in signaling pathways that activate defense mechanisms. However, excessive ROS can be detrimental to plant cells, causing oxidative damage. GABA application in plants induces a metabolic pathway to modulate ROS levels in response to biotic stress, thereby maintaining a balance between effective defense responses and cellular protection. Herein, GABA treatment in wheat plants significantly enhanced the hypersensitive response by increasing ROS levels, particularly pre-/post-GABA-treated plants. This resulted in a stronger oxidative burst in both susceptible and moderately resistant cultivars against leaf rust infection. Specifically, GABA elevated O_2_^●−^ levels in guard cells and surrounding stomata, promoting stomatal closure and the hypersensitive response (Figure 3 and Figure 4). In the susceptible cultivar, O_2_^●−^ accumulation was higher upon infection with *P. triticina* compared to the moderately resistant cultivar. Furthermore, GABA-treated plants consistently exhibited elevated O_2_^●−^ levels compared to mock-treated controls. The hydrogen peroxide (H_2_O_2_) production was also detected in the mesophyll cells of both treated and untreated cultivars, indicating a constitutive defense mechanism. The Sakha 94 cultivar showed higher H_2_O_2_ levels upon infection, suggesting a robust defense response (Figure 3 and Figure 4). The accumulation of H_2_O_2_ in guard and mesophyll cells further contributed to stomatal closure and the hypersensitive response. These findings underscore GABA’s role in modulating plant defense mechanisms by enhancing ROS production, thereby improving resistance to leaf rust disease. Numerous studies have demonstrated the significant impact of GABA on the production of ROS, particularly O_2_^●−^ and H_2_O_2_. Research across various plant species reviewed GABA’s role in enhancing ROS levels, which are crucial for activating defense mechanisms [22,24,44]. In barley, GABA treatment led to increased ROS production, contributing to a robust hypersensitive response and alleviating oxidative damage caused by aluminum stress [45]. Similarly, GABA elevated H_2_O_2_ levels, enhancing the activation of antioxidant defenses while mitigating the impact of *Sogatella furcifera* in rice [43] and *Colletotrichum fructicola* in apple [46]. Overall, these studies, including our investigation, collectively underscore the crucial role of GABA in enhancing ROS production.

In this study, GABA treatment effectively enhanced the accumulation of soluble phenols in both wheat cultivars. Phenolic compounds play a crucial role in plant defense mechanisms, and their increased presence suggests a stronger protective response [47]. Our results revealed that infection with *Pt* significantly increased the phenolic content in both cultivars, regardless of the GABA treatment. In the Morocco cultivar, the phenolic levels in infected plants ranged from 120 to 140 µg/g FW across all treatments, with no significant differences between mock-treated and GABA-treated plants (Figure 5). This indicates that the infection itself was a primary factor in elevating phenolic content. Interestingly, pre-GABA- and pre-/post-GABA-treated uninfected plants showed slightly higher phenolic concentrations compared to mock-treated uninfected plants, suggesting that GABA treatment may have a priming effect, preparing the plant for potential pathogen attack. For the Sakha 94 cultivar, a similar trend was observed, but with more pronounced effects of GABA treatment. The highest phenolic levels were found in pre-/post-GABA-treated plants, followed by pre-GABA- and mock-inoculated plants, respectively.

In the presence of GABA, the antioxidant enzymes CAT and POX play a critical role in managing ROS. The application of GABA in plants enhances CAT and POX activities, facilitating the decomposition of H_2_O_2_ into H_2_O and O_2_, thereby reducing ROS levels and mitigating oxidative stress [42,48]. This mechanism helps protect plant cells from ROS-induced damage, contributing to improved plant resilience against various stresses [49]. Furthermore, antioxidant enzymes are vital in mitigating oxidative stress induced by pathogen attacks, and their increased activity following GABA treatment highlights the potential of GABA as a protective agent [46]. Herein, exogenous GABA has a notable impact on CAT and POX enzyme activities in wheat cultivars, enhancing the plant’s defense response to fungal infection (Figure 5). For CAT activity, GABA treatment led to a marked increase in both cultivars, with the most pronounced effects observed in infected Sakha 94 plants. Infected Morocco plants treated with pre-/post-GABA exhibited the highest CAT activity, compared to lower activity levels in pre-GABA- and mock-treated plants. Uninfected Morocco plants displayed significantly lower CAT activity, indicating that infection, coupled with GABA treatment, significantly enhances CAT activity. Similarly, in the Sakha 94 cultivar, infected plants showed high CAT activity levels, particularly in pre-/post-GABA-treated plants. This contrasted with uninfected plants, where CAT activity was lower. The effects of GABA on POX activity mirrored those observed for CAT. In both cultivars, GABA treatment significantly increased POX activity, especially in infected plants. Infected Sakha 94 plants had the highest POX activity levels, with pre-/post-GABA and pre-GABA treatments leading to the most significant increases. Uninfected plants from both cultivars exhibited lower POX activity, with GABA-treated plants showing slightly higher activity than mock-treated plants. These findings attested to the effectiveness of GABA in elevating CAT and POX activities and improving oxidative stress management, even in susceptible cultivars. A similar study on tomato plants revealed the impact of GABA in enhancing resistance against Alternaria rot caused by *Alternaria alternate* via activation of catalase for managing secondary metabolites [50]. This enhanced enzymatic activity is crucial for mitigating oxidative damage and supporting the plant’s defense mechanisms against fungal pathogens.

## 4. Materials and Methods

### 4.1. Leaf Rust Fungal Race

The virulent race of wheat leaf rust, *P. triticina* (*Pt*), race PTKTT, was obtained from the Wheat Disease Research Department, Plant Pathology Research Institute, Agricultural Research Centre, Egypt. The race was maintained on the susceptible wheat (*T. aestivum*) cultivar, Morocco, under controlled conditions to ensure its viability and pathogenicity. Prior to inoculation experiments, the leaf rust race was propagated on wheat seedlings in a growth chamber set to conducive conditions for rust development, including a temperature range of 20–22 °C and high humidity levels (>90%). The infected wheat plants served as a continual source of spores for subsequent inoculation trials.

### 4.2. Selected Wheat Cultivars

Two wheat cultivars, named Morocco and Sakha 94, the susceptible and moderately resistant to leaf rust race PTKTT, respectively, were sourced from the Wheat Research Section, Field Crop Institute, Agriculture Research Centre, Cairo, Egypt. The grains underwent careful inspection to ensure uniformity, with any damaged or irregular grains being removed from the batch. The grains were separated and sown in plastic pots (10 cm in diameter) filled with soil. Each pot accommodated 3 grains, ensuring appropriate spacing for growth. Subsequently, the pots were transferred to a growth chamber situated in a greenhouse where temperatures were maintained within range from 20 to 22 °C. Irrigation was administered consistently to the plants to maintain the optimal moisture levels essential for their development.

### 4.3. Experimental Design and GABA Treatment

**Figure 6:**
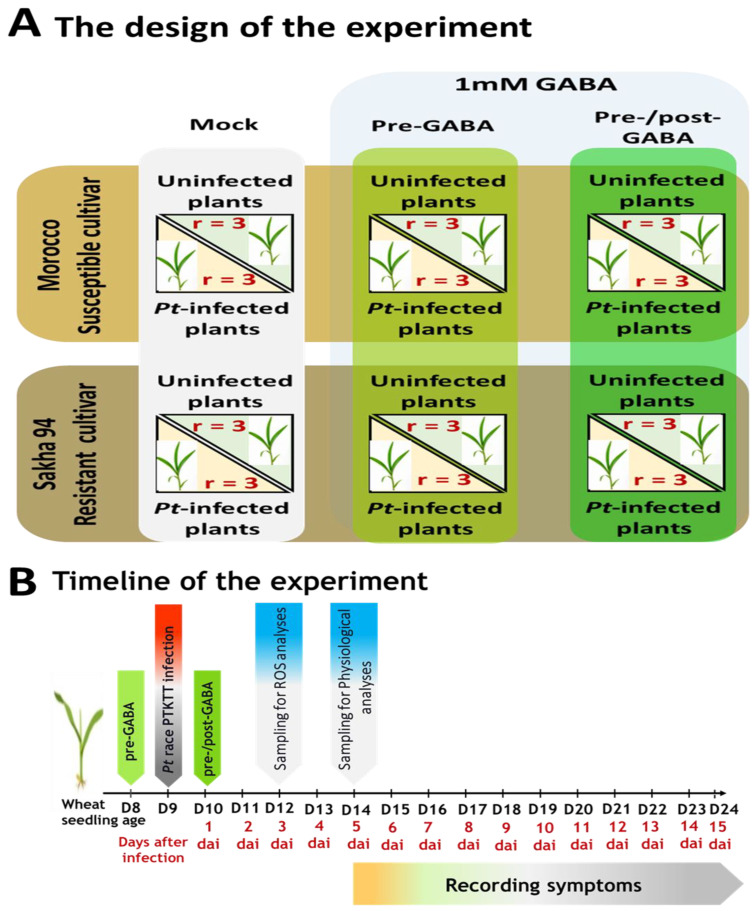
Experimental design and timeline for assessing the effectiveness of GABA in enhancing resistance to leaf rust in wheat cultivars. (**A**) The experiment included Morocco and Sakha 94, the susceptible and resistant wheat cultivars to leaf rust (PTKTT) fungal race. The plants were divided into two groups: uninfected and *Pt*-infected plants. The mock-treated plants, H_2_O foliar-sprayed plants; pre-GABA, plants received 1 mM GABA treatment, as a foliar spray, twenty-four hours prior to infection; pre-/post-GABA, plants received 1 mM GABA treatment both 24 h before and after infection. The experiment was performed in triplicates, within each group comprising three random replicates consisting of three pots, and each pot contained three plants. (**B**) Timeline of the entire experiment, including the time of GABA treatments, infection, sampling at 3 dai and 5 dai, and symptom record.

The efficacy of GABA in alleviating the detrimental effects induced by *Pt* fungus, race PTKTT, on selected wheat cultivars was assessed, including both those exhibiting susceptibility and moderate resistance to the fungal pathogen. The impact of spraying wheat-seedling leaves with 1 mM GABA on leaf rust disease symptoms and infection types induced by race PTKTT on both Morocco and Sakha 94 wheat cultivars was examined by a completely randomized experiment designed as depicted in Figure 6. The experimental design involved the division of plants into two primary treatment groups, each receiving a foliar spray of 1 mM GABA based on previous pervious investigations [33,42]. The first treatment was administered twenty-four hours prior to pathogen infection (pre-GABA), while the second treatment was applied both twenty-four hours before and after the infection (pre-/post-GABA). Additionally, a control group was established, consisting of plants treated with a mock solution of distilled water. Within each main group, the plants were further categorized into two subgroups: those infected with the pathogen and those that remained uninfected. Each subgroup comprised three plant replicates, ensuring a robust experimental framework for assessing the effects of GABA on plant response to infection. The experiment was performed in triplicates: within each group, there were three random replicates consisting of three pots, and each pot contained three plants. Infection types (ITs) were recorded to track disease progression. For this, the first leaves of the wheat were subjected to visual inspection at regular intervals following inoculation, unveiling discernible patterns in the development of rust symptoms.

### 4.4. Rust Inoculation

Original rust-infected seedlings, harboring the race PTKTT, were brushed against the tested seedlings and gently shaken over them. Following this, the inoculated plants were promptly sprayed again with tap water to create an initial film of water on the foliage. The inoculated seedlings were then placed in dark, humid chambers maintained at 18–20 °C and 100% relative humidity for 24 h. This facilitated stomatal opening through transpiration, ensuring uniform pathogen germination across all infected samples, thereby promoting leaf rust development in wheat [51]. Afterward, the inoculated seedlings were transferred to their respective benches in the greenhouse and monitored for 15 days, at a temperature of 20 ± 2 °C, with a relative humidity of 50–55%.

### 4.5. Sampling

The sampling procedure encompassed the collection of wheat seedlings’ first leaves at two time points: three days and five days post-inoculation (Figure 6). These samples were subsequently utilized for histochemical and physiological analyses, allowing for a detailed examination of the plants’ responses to the inoculation over time.

### 4.6. Symptom Records

The first leaves underwent systematic visual examination for rust symptoms at regular intervals post-inoculation. Disease symptoms were documented on wheat leaves based on infection types (ITs), according to established criteria, with each observed symptom corresponding to a specific IT classification [52]. Infection types, namely 0, ;, 1, and 2, were categorized as resistant (R), while 3 and 4 were classified as moderately susceptible (MS) and susceptible (S), respectively. Additionally, the density of pustules per square centimeter on the upper leaf surface was quantified following the methodology described by Menzies and Belanger [53]. Symptoms were recorded, considering their severity and distribution, to evaluate disease progression over time.

### 4.7. Incubation and Latent Periods

The incubation period (IP) was determined by counting the number of visible pustules on marked leaves per day until no more pustules developed. The latent period (LP) was determined by the time between inoculation and 50% of pustules that were evident or emerged, according to Parlevliet and Kuiper [54].

### 4.8. Pustule Size Measurements

Pustule size was determined using the software of the employed light microscope (Leica DM2500, Wetzlar, Germany). As described by Kemen [55], leaves were collected 20 days post-inoculation, and pustules were fixed in acetic acid/ethanol solution (1:3, *v*/*v*), cleared using a mixture of preheated (95 °C) lactophenol/ethanol solution (1:2, *v*/*v*), and subsequently stained with 0.1% trypan blue. Following incubation in boiling potassium hydroxide (0.125 M) for 1 to 3 min, samples were mounted in 100% glycerol. Measurements were conducted on at least 20 randomly selected pustules per leaf.

### 4.9. Stomatal Closure Assessment

Twenty-one-day-old wheat plants were used for this investigation and were cultivated under precisely controlled environmental conditions within a growth chamber. These conditions included a constant temperature of 20 °C and a photoperiod of 16 h of light followed by 8 h of darkness. Prior to treatment, the plants underwent a 24-h dark period to induce stomatal closure. Following the dark treatment, the plants were randomly assigned to two experimental groups: a treatment group receiving foliar application of 1 mM GABA solution and an untreated control group receiving foliar application of water (mock treatment). The foliar treatments were administered using a fine-mist sprayer to ensure uniform coverage across the apical first leaves of each plant. Subsequently, after a three-hour treatment application period, the apical first leaves were carefully excised and mounted on glass slides. Stomatal apertures were visualized under a light microscope (Olympus, DS50, Tokyo, Japan) with 40× magnification settings. Digital images of stomata were captured using a digital camera attached to the microscope, ensuring consistent settings across all samples. Stomatal aperture measurements were conducted using image analysis software (imageJ; [56]), allowing for precise quantification of stomatal opening. The experiment was performed in triplicate to guarantee the reliability of the results. Statistical analysis was performed on 20 stomata for each group.

### 4.10. Histochemical Analysis of Reactive Oxygen Species (ROS)

The detection of superoxide anion (O_2_^●−^) and hydrogen peroxide (H_2_O_2_) was carried out using the nitro blue tetrazolium (NBT) and 3,3-diaminobenzidine (DAB) staining methods, respectively, following the protocol described by Thordal-Christensen et al. [57]. Eight hours after the first sampling time (3 dai), the first seeding leaves were collected and incubated with a solution comprising 10 mM sodium azide and 10 mM potassium phosphate buffer (pH 7.8), supplemented with 1 mg/mL NBT or DAB sourced from Sigma-Aldrich, Missouri, USA. After that, an additional 8-h incubation period was added to facilitate uptake and reaction with O_2_^●−^ and H_2_O_2_. Segments of about 3 cm were excised from the center of the inoculated leaves. These leaf sections were then fixed, decolorized by boiling in 95% ethanol for 10 min, and cleared in saturated chloral hydrate. The treated leaf segments were preserved in a microscopy solution (50% glycerol). For microscopic analysis, the cleared leaf segments were mounted on glass slides in microscopy solution, examined, and photographed using a light microscope (Leica, DM 2500).

### 4.11. Assay of Total Phenols

The total phenol concentration was quantified using the Folin–Ciocâlteu method, with catechol as the standard, following the protocol described by Singleton and Rossi [58]. In this procedure, 100 μL of each sample extract was diluted to 3 mL with distilled water, and then 0.5 mL of Folin–Ciocâlteu reagent was added. After a 3-min incubation, 2 mL of a 20% sodium carbonate solution was introduced, and the mixture was thoroughly mixed. The color development was allowed to proceed for 60 min before measuring the absorbance at 650 nm using a CT 200 spectrophotometer, Hüttenberg, Germany. Calibration curves were generated using various concentrations of catechol solution (5 mg/100 mL), and the results were expressed as milligrams of catechol per 100 g of fresh-weight material.

### 4.12. Assay of Total Soluble Protein Content

Total soluble protein quantification was performed using the Bradford assay. The method was described by Bradford [59]. The assay involved mixing 100 μL of crude enzyme extract with 2.9 mL of Bradford reagent, followed by a brief vortexing and then resting via a 5-minute incubation at room temperature to allow the formation of the protein–dye complex. Absorbance was measured at 595 nm using a UV-Vis spectrophotometer (UV 9100 B, LabTech, Hopkinton, MA, USA). A standard curve was established using bovine serum albumin (BSA) in concentrations ranging from 0 to 10 mg/mL, with absorbance values plotted against these concentrations to determine the protein content in the samples.

### 4.13. Enzyme Analyses

Assay of catalase activity: The activity of catalase was measured following the method described by Aebi [60]. An amount of 100 μL of enzyme extract was combined with 2.9 mL of a reaction mixture (20 mM H_2_O_2_ and 50 mM sodium phosphate buffer, pH 7.0). Catalase activity was determined by monitoring the decrease in absorbance at 240 nm due to H_2_O_2_ consumption. The consumed H_2_O_2_ quantity was calculated using a molar extinction coefficient of 0.04 cm^2^/μmol. One unit of enzyme activity was defined as the decomposition of 1 μmol of H_2_O_2_ per minute. Catalase activity was detected as units per minute per milligram of protein.

Assay of peroxidase activity: The peroxidase activity was assayed using the method described by Hammerschmidt et al. [61]. An approximate 2.9 mL from the reaction mixture containing 0.25% (*v*/*v*) o-methoxyphenol (guaiacol) in 10 mM sodium phosphate buffer, pH 6, involved 10 mM H_2_O_2_. To initiate the reaction, 100 μL of the crude enzyme extract was added, and the reaction was monitored spectrophotometrically using a CT 200 spectrophotometer at 470 nm per minute. Enzyme activity was quantified as one international unit (IU), defined as the amount of enzyme catalyzing the conversion of 0.01 OD units of guaiacol per minute per milligram of protein.

### 4.14. Statistical Analysis

All data were analyzed using SPSS version 14, the comprehensive software for statistical analysis. The experiments were performed in triplicates, with each treatment group consisting of three plants. Statistical analysis was conducted using a one-way ANOVA to assess the significance, and a post hoc Tukey test was applied for multiple comparisons between treatments. The level of significance was set at *p*-value < 0.05, ensuring that the differences observed between the treatments were statistically valid.

## 5. Conclusions

The increasing prevalence of wheat leaf rust, caused by *P. triticina*, highlights the shortcomings of conventional disease management strategies. To develop sustainable solutions, it is essential to address genetic, agronomic, and ecological factors; however, challenges such as rapid pathogen evolution and the necessity for adaptable breeding strategies remain significant obstacles. This study evaluated the efficacy of GABA in enhancing resistance to leaf rust in wheat cultivars with different levels of inherent resistance, focusing on the susceptible Morocco and the moderately resistant Sakha 94. Our findings contribute to a growing body of evidence that supports the potential of GABA as a biostimulant for improving plant defense mechanisms. Through a completely randomized design, the study assessed the impact of exogenous GABA treatment on both infected and uninfected plants challenged with the leaf rust fungal race PTKTT. The results demonstrated that GABA significantly enhanced resistance in both Morocco and Sakha 94 cultivars. Notably, GABA treatment in the Morocco cultivar reduced the infection type and decreased pustule densities and sizes, while also extending the incubation and latent periods of the disease. These outcomes underscore GABA’s potential as a tool for controlling leaf rust, particularly in cultivars with lower inherent resistance. Furthermore, GABA treatment was shown to influence stomatal behavior by inducing significant stomatal closure, particularly under dark conditions. This stomatal regulation may enhance the plant’s resilience to both biotic and abiotic stresses by reducing the risk of fungal infections. The study also highlighted GABA’s role in modulating ROS production, thereby enhancing the plant’s hypersensitive response and overall resistance to leaf rust. The application of GABA not only enhances phenolic accumulation and antioxidant enzyme activities but also contributes to better oxidative stress management and improved plant defense responses. Overall, this study represents the first report on the impact of GABA on leaf rust disease in wheat. The findings suggest that GABA could be a valuable addition to wheat disease management strategies, offering benefits across different cultivars.

## 6. Perspectives

To advance our understanding of the role of GABA in improving wheat resistance to leaf rust, several critical areas for future research warrant exploration. Firstly, it is essential to identify and characterize the specific transporters involved in the delivery of GABA within wheat plants. Gaining insights into the transporters that facilitate GABA uptake and distribution, particularly during pathogen infection, will be instrumental in optimizing GABA application strategies. Additionally, research should focus on determining the most effective delivery methods for GABA to maximize disease resistance. This includes evaluating various application techniques to assess their efficacy in ensuring that GABA reaches critical sites within the plant and exerts its protective effects. Furthermore, investigating the potential synergistic effects of GABA in combination with other treatments, such as priming agents, is recommended. GABA has been identified as a safe and effective treatment for enhancing grain yield, particularly under stress conditions. Research indicates that GABA application can significantly improve various yield-related traits, including panicle length, total tillers, and the number of grains per panicle [62]. The safety of GABA application is supported by its role in modulating plant stress responses without adverse effects on plant health, making it a promising tool for sustainable agriculture aimed at improving food security. These integrated approaches could enhance GABA’s influence on overall plant defense mechanisms. Collectively, these studies may lead to the development of more effective and sustainable strategies for managing wheat leaf rust, ultimately improving crop resilience.

## Figures and Tables

**Table 1:** Comparative symptom measurements in the wheat Morocco and Sakha 94 cultivars inoculated with *Pt* fungus and treated with 1 mM GABA.

Symptom Measurements	Mock		Pre-GABA Application		Pre-/Post-GABA Application	
	**Morocco**	**Sakha 94**	**Morocco**	**Sakha 94**	**Morocco**	**Sakha 94**
Infection type	4	3	3	2	2	1
No. of pustules/ (cm^2^)	32.88 ^a^	23.42 ^b^	24 ^b^	17.83 ^c^	12.06 ^d^	9.16 ^d^
Pustules size (mm^2^)	0.947 ^a^	0.743 ^b^	0.586 ^c^	0.393 ^d^	0.356 ^d^	0.253 ^e^
Incubation period (IP)	6.25 ^d^	8.58 ^c^	7.78 ^c^	11.18 ^b^	11.18 ^b^	13.2 ^a^
Latent period (IP)	11.27 ^e^	14.22 ^cd^	13.15 ^d^	15.3 ^bc^	15.95 ^b^	20.01 ^a^

Morocco = susceptible cultivar to PTKTT; Sakha 94 = moderately resistant cultivar to PTKTT. Different letters above the bars indicate statistically significant differences (*p* < 0.05) among treatments within each group, according to Tukey test.

## Data Availability

All relevant data are included within this article.
